# Transient Thyroiditis after Surgery for Tertiary Hyperparathyroidism: A Case Report

**DOI:** 10.3389/fendo.2015.00123

**Published:** 2015-08-18

**Authors:** Yasser Ali Hakami

**Affiliations:** ^1^Obesity, Endocrine and Metabolism Center, King Fahad Medical City, Riyadh, Saudi Arabia

**Keywords:** thyroiditis, thyroid function tests, hyperparathyroidism, post-operative period, parathyroidectomy, adrenergic beta-antagonists

## Abstract

Parathyroid (PTH) exploration surgery carries the risk of developing post-operative thyroiditis due to vigorous manual manipulation of the thyroid gland during surgery. Post-operative thyroiditis has a wide spectrum of clinical manifestations. However, it remains underreported. Here, we describe a case of post-operative transient thyroiditis in a 33-year-old male who developed 3 days after parathyroidectomy for PTH hyperplasia. We review the limited literature regarding this interesting entity.

## Introduction

Post-operative thyroiditis is one cause of iatrogenic thyrotoxicosis. It usually occurs after vigorous manual manipulation of the thyroid gland during neck or parathyroid (PTH) exploratory surgery. It has a wide spectrum of clinical manifestations, varying from asymptomatic hyperthyroxinemia to a severe overt thyrotoxic state (1).

However, this disorder remains underestimated and only a few cases have been reported. In current clinical practice, this condition is likely more common and important than previously thought.

## Case Report

A 33-year-old male came to the clinic complaining of generalized bone pain associated with progressive weakness over the last year. The patient had been diagnosed with end-stage renal disease secondary to glomerulonephritis 11 years before his presentation and had been on hemodialysis three times per week for the last 10 years. The patient had no past history of thyroid disorders. There was no goiter on physical examination.

The patient’s serum corrected-calcium level was 2.79 mmol/L (normal range, 2.19–2.54 mmol/L), his serum phosphate level was 1.29 mmol/L (normal range, 0.7–1.5 mmol/L), his vitamin D25-OH level was normal, his serum creatinine level was 756 μmol/L (normal range, 53–106.1 μmol/L), his serum urea was 17.2 mmol/L (normal range, 2.9–8.2 mmol/L), and his serum PTH level was 489.5 pmol/L (normal range, 1.6–6.8 pmol/L).

**Figure 1:**
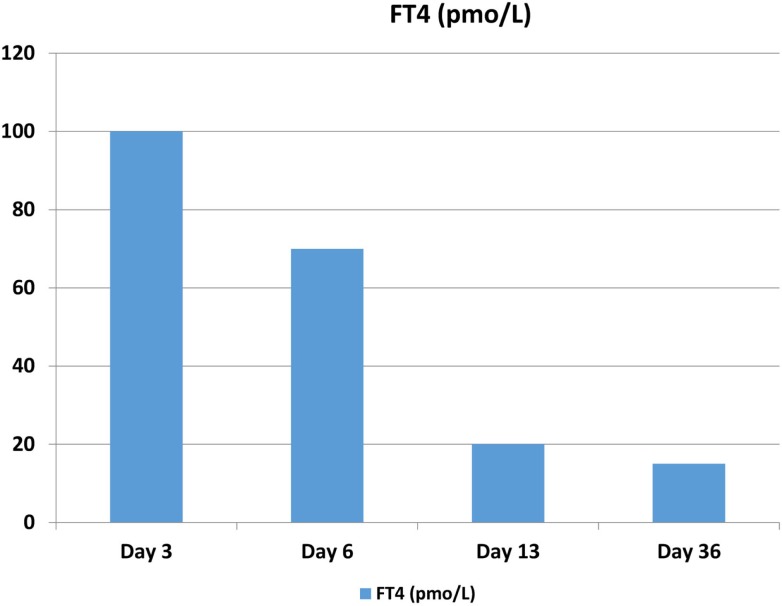
**The patient’s FT4 levels peaked on post-operative day 3**. Reference values are the following: free thyroxine (FT4): 9.1–23.8 pmol/L.

At our clinic, the patient was diagnosed with tertiary hyperparathyroidism and renal osteodystrophy as complications from his chronic kidney disease. His pre-operative serum thyroid-stimulating hormone (TSH) level was within normal parameters (1.1 mIU/L; normal range, 0.35–5.0 mIU/L). A dual-phase PTH scan revealed two foci located below the lower poles of both thyroid lobes with intact thyroid uptake compatible with PTH adenomas (Figure 1). Diagnostic computed tomography (CT) or magnetic resonance imaging (MRI) scans were necessary for the final reading, which involved fusion of the available axial cuts of the PTH scan with structural imaging data. Neck MRI revealed four PTH masses posterior and inferior to the normal thyroid gland, with two masses on each side, suggesting PTH adenomas. Fine-needle aspiration (FNA) of the right PTH masses was performed, and pathological examination revealed PTH cells consistent with hyperplastic proliferation. Therefore, the patient was diagnosed with PTH hyperplasia.

The patient underwent parathyroidectomy to remove his PTH glands. He was brought to the operating room under general anesthesia. A transverse incision 6-cm long and 3-cm superior to the sternal angle was made. The strap muscle was separated, and the dissection was continued until the surgeon reached the thyroid gland. Retraction of the thyroid gland on the right side was successful, and the recurrent laryngeal nerve was identified. The superior right PTH gland was anterior to the nerve and was dissected successfully. The inferior right PTH gland was deep and near the sternum. It was difficult to separate the inferior right PTH gland from the thyroid gland surgically because they were firmly attached, so the attached part of the thyroid gland was taken as a resection margin along with the inferior right PTH gland. Subcapsular dissection of the thyroid gland was done on the left side also, and the left recurrent laryngeal nerve was identified. The superior left PTH gland, posterior to the nerve, was identified and separated successfully. The left inferior PTH gland was located, dissected from around the gland, and extracted. All of the PTH glands were resected. The duration of the surgery was 78 min.

The intra-operative serum PTH level was 414.2 pmol/L and had declined to 22.4 pmol/L 1 h after surgery. One day after surgery, it was 4.2 pmol/L. The resected part of the thyroid gland was normal on pathological examination. The pathological examination of the resected PTH glands revealed nodular hyperplasia involving all glands confirming the PTH hyperplasia diagnosis. There were no complications during surgery. However, the patient developed hypocalcemia that was normalized with calcium replacement therapy.

**Figure 2:**
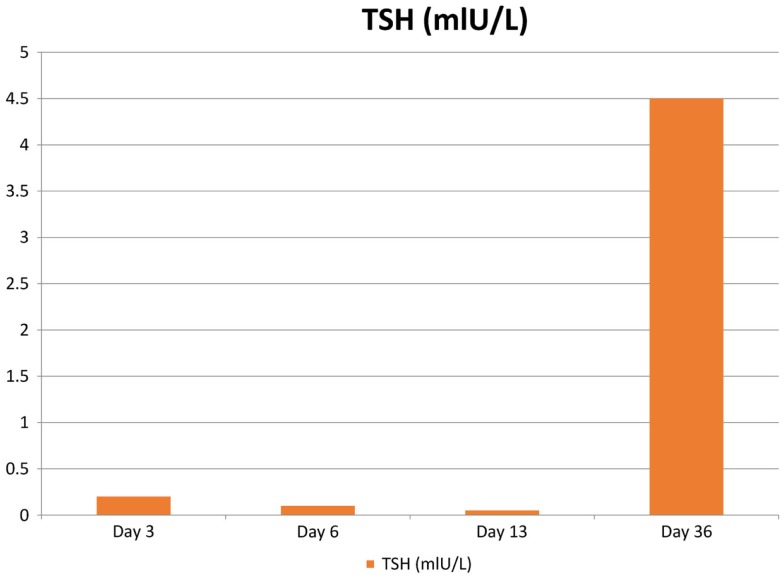
**The patient’s TSH levels following surgery**. Reference values are the following: thyroid-stimulating hormone (TSH): 0.35–5.0 mIU/L.

**Figure 3:**
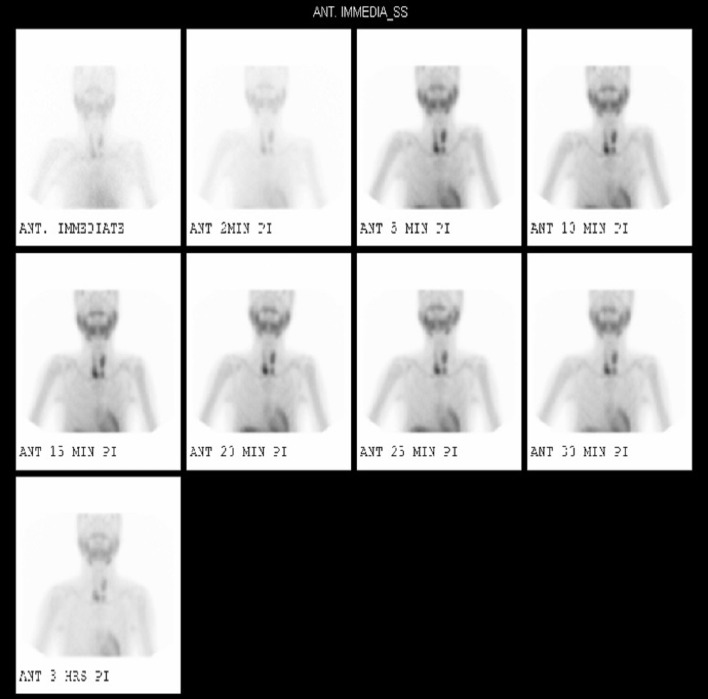
**99m TcMIBI (sestamibi) dual-phase parathyroid scintigraphy revealed two foci located below the lower poles of both thyroid lobes with intact thyroid uptake compatible with parathyroid adenomas**.

**Figure 4:**
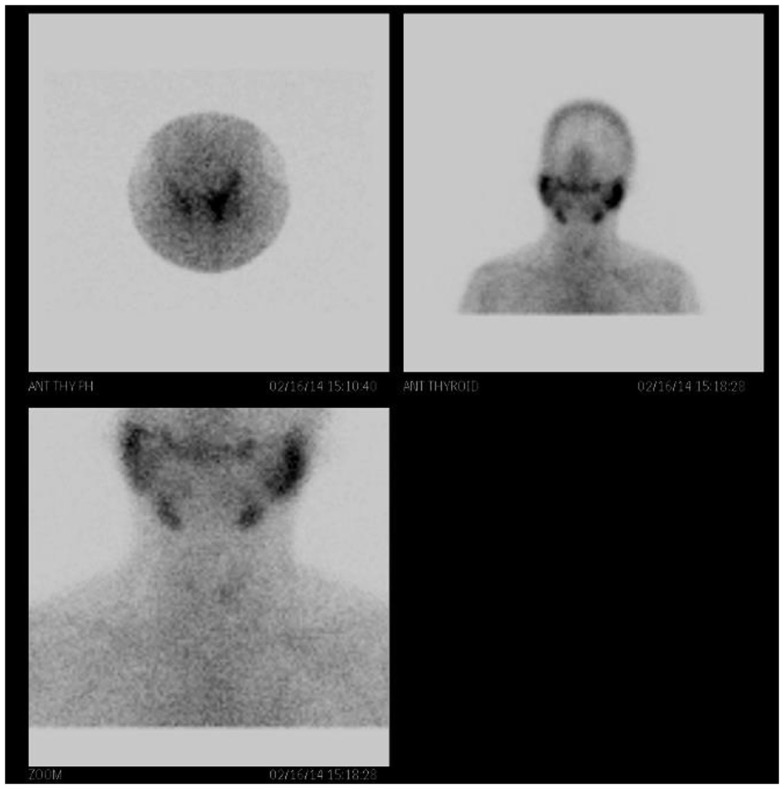
**Technetium-99m (Tc-99m) pertechnetate scintigraphy of the patient’s thyroid gland is faintly observed with generalized reduced tracer uptake in the thyroid gland**. There is increased background tracer activity in the salivary glands. These findings are compatible with thyroiditis.

Three days after the surgery, the patient presented with new symptoms of palpitation, anxiety, and hand tremors. A complete examination revealed mild, diffuse, non-tender goiter, and fine tremor, with stable vital signs. Serum thyroid function tests revealed that his TSH level was 0.171 mIU/L and that his free thyroxine (FT4) level was 97.6 pmol/L (normal range, 9.1–23.8 pmol/L), consistent with primary hyperthyroidism (Figures 2 and 3). Technetium-99m (Tc-99m) pertechnetate scintigraphy revealed generalized reduced tracer uptake in the thyroid gland, a finding that is compatible with thyroiditis (Figure 4). Autoimmune markers were normal, including thyroglobulin (Tg) antibodies (2.08 IU/mL; normal range, 0–80 IU/mL), thyroid peroxidase (TPO) antibodies (0.3 IU/mL; normal range, 0–20 IU/mL), and TSH receptor antibodies (negative). However, inflammatory markers were elevated including C-reactive protein (CRP) (8.8 mg/L; normal range, 1.0–3.0 mg/L) and erythrocyte sedimentation rate (ESR) (78 mm/H; normal range, 0.0–20.0 mm/H). There was no clinical evidence of sepsis. The patient did not receive iodinated contrast agents; he did not receive lithium or amidarone nor medication that might have affected his thyroid function tests. Other possible causes of thyroiditis have been ruled out, specifically, viral thyroiditis, bacterial thyroiditis, autoimmune thyroiditis, radiation thyroiditis, or drug-induced thyroiditis. Therefore, he was diagnosed with post-operative thyroiditis and given oral Bisoprolol. He did not receive an antithyroid agent.

His condition improved over the following weeks. After 3 weeks, he made a complete recovery and had no persistent clinical or biochemical manifestations of thyroid disease. Bisoprolol was withdrawn. The patient was examined in the clinic after 6 months. At this time, he was clinically and biochemically euthyroid, with no indication of hypothyroidism.

A definitive diagnosis of post-operative transient thyroiditis secondary due to manual manipulation of the thyroid gland during surgery was made.

## Discussion

The incidence of post-operative thyroiditis is unknown as it is underreported. There are no data in the literature pertaining to complications of parathyroidectomy on the prevalence of post-operative transient thyroiditis. This is likely because most patients are asymptomatic or have mild non-specific symptoms.

In 2005, Stang et al. examined pre- and post-operative thyroid function and outcomes in 199 patients who had PTH exploratory surgery for primary sporadic hyperparathyroidism. They found that the incidence of TSH suppression below the lower limits of normal levels was 29% (58/199) (1). Akira et al. reported a type of thyrotoxicosis that occurred after needle aspiration of thyroid cysts. The incidence of post-aspiration thyrotoxicosis was 0.9% (1 of 115) (2). In two small studies, the rate of post-operative hyperthyroidism after parathyroidectomy was 20% (3, 4). Reported causes of iatrogenic thyrotoxicosis include use of medication that can affect the thyroid gland, such as lithium and amiodarone, recent radioiodine ablation, thyroid hormone replacement therapy, iodine excess, immunotherapy, external irradiation, and manipulation of the thyroid gland during surgery.

The exact mechanism of post-operative thyroiditis is unclear. In the past, hyperthyroidism arising after PTH surgery has been thought to be transient and relate to retraction of the thyroid gland for exposure during surgery.

In 1975, Carney et al. described an association between vigorous thyroid lobe palpations under pentobarbital anesthesia with the histological finding of granulomatous thyroid folliculitis in massaged thyroid lobes in dogs. Noting that similar multifocal inflammatory changes usually were present in resected human thyroid lobes. It is suggested that manipulation of the thyroid gland was sufficient to cause transient inflammatory reactions in both humans and dogs, which they termed as palpation thyroiditis and multifocal granulomatous folliculitis (5).

In 1992, Walfish et al. reported on three patients who had undergone resection of PTH adenoma, which occurred after routine transection of the superior thyroid pole and vessels and developed thyrotoxicosis post-operatively. They concluded that the thyroid disruption caused post-operative hyperthyroidism. They were the first to suggest evidence of a clinical syndrome of trauma-induced thyroiditis in those patients (6). On the contrary, in Stang et al. study, they treat the thyroid gland as gently as possible, but there data supported a causative role of operative trauma to the thyroid lobe, since bilateral exploration was associated with the development of post-operative hyperthyroidism (1).

In Akira et al. study, the mechanism of post-aspiration thyrotoxicosis is unknown; however, they speculated that the combination of thyroiditis and leakage of thyroid contents into the cyst might have triggered thyroid hormone release into circulation after needle aspiration of thyroid cysts (2). In Bergenfelz et al. prospective study of 20 patients undergoing operation for primary hyperparathyroidism, compared with 6 patients undergoing laparoscopic cholecystectomy. Four patients (20%) developed biochemical hyperthyroidism, but serum antithyroid antibody levels did not increase. Control patients experienced a slight increase in mean TSH post-operatively, and the authors concluded that post-parathyroidectomy hyperthyroidism was related to metabolic derangement intrinsic to the primary hyperparathyroidism itself (3).

In Lindblom et al. report, they examined post-parathyroidectomy hyperthyroidism in 26 patients undergoing operation for primary hyperparathyroidism, but with more detail about anesthetic and operative factors and with a different control group of 11 patients undergoing breast cancer surgery. Post-operative levels of T4 and T3 were higher after PTH operation than after breast operation. The investigator concluded that manipulation of the thyroid gland was most likely the major contributing factor to post-operative hyperthyroidism. However, it may not be the sole explanation, since there data suggest a more multifactorial scenario (4).

Rudofsky et al. reported a case of transient symptomatic thyrotoxicosis following PTH surgery for tertiary hyperparathyroidism in a 33-year-old woman; they presumed that it was caused by a traumatic thyroiditis as a result of manipulation of the thyroid gland during surgery (7).

Calle and Cohen reported on a patient who developed transient thyroiditis following total laryngopharyngoesophagectomy with gastric pull-up for a right hypopharynx tumor with preservation of the thyroid gland (8).

In the Stang et al. study cited above, the incidence of post-operative hyperthyroidism was independent of age, gender, the pre-operative and intra-operative PTH levels, and adenoma weight. There was no relationship between the type of PTH pathology, that is, multi-glandular disease versus solitary adenoma, the presence of goiter, or prior thyroiditis. None of the following indicators of operative trauma were predictive of post-operative thyroiditis: time that the operation took, incision length, difficulty in making the dissection, thyroid nodulectomy, thyroidectomy for intrathyroidal PTH adenoma, retropharyngeal adenoma, concurrent cervical thymectomy, the degree of perithyroidal inflammation, or whether or not a drain was placed (1).

Stang et al. identified three surgical variables associated with post-parathyroidectomy hyperthyroidism. These were bilateral exploration, the absence of concurrent thyroid lobectomy, and performance of the operation at community hospitals. When patients were cared for at the community hospitals, exploration had to be done bilaterally because the intra-operative PTH could not be monitored. The use of lithium was observed to be an independent risk factor for post-operative hyperthyroidism. This may be because lithium provides a persistent toxic insult to thyroid tissue, rendering it more sensitive to manipulation during neck surgery. Patients who underwent complete thyroid lobectomy were less likely to have post-operative thyroiditis (1).

Post-operative thyroiditis has a wide clinical presentation spectrum, ranging from asymptomatic hyperthyroxinemia to severe hyperthyroidism. In addition, a few cases of thyroiditis following neck surgery that caused the onset of atrial fibrillation have been reported (9, 10). McDermott et al. reported a case of transient thyroiditis secondary to surgical manipulation of the thyroid in a 76-year-old man, which caused significant tachyarrhythmia following total laryngectomy for a subglottic tumor with preservation of thyroid gland (11).

In the Stang et al. study, hyperthyroidism symptoms were reported in 19 of 125 (15%) patents with pre-operatively normal serum TSH levels. Of these, five patients (4%) were found to be overtly thyrotoxic. The duration for complete biochemical resolution ranged from 12 days to 90 days post-operation, whether or not there was medical management (1). The finding that there was spontaneous resolution of the condition within 6 weeks in the majority of the patients supports the hypothesis that manipulation of the gland during parathyroidectomy is a major contributing factor in its development. Development of thyrotoxicosis with more prolonged hyperthyroxinemia later than 6 weeks after surgery may be caused by other factors, including lithium therapy or a predisposition to Grave’s disease (12). It is not clear that permanent hypothyroidism is common in these cases, as there are few data on this question. There is also little information on how an abnormal thyroid status will affect the development of post-operative thyroiditis compared to patients with a normal thyroid. Nonetheless, it would appear to be prudent to follow up patients who suffered post-operative thyroiditis for permanent hypothyroidism. In all cases, symptomatic treatment should be applied until spontaneous resolution of symptoms.

We speculate that there is an association between vigorous manual manipulation of the thyroid gland during PTH exploration surgery and the incidence of post-operative transient thyroiditis.

Further retrospective and prospective studies should be conducted to confirm the association between vigorous manual manipulation of the thyroid gland during PTH exploration surgery and the incidence of post-operative thyroiditis. They should also address identification of the risk factors and the patients who should undergo additional testing. This would be important to enhance our understanding of the disease process, and establish the clinical criteria for accurate diagnosis and early treatment.

Post-operative thyroiditis can cause significant clinical problems that can be easily missed. Clinicians should have an increased awareness of the clinical consequences and complications.

Establishing the risks inherent to PTH exploration surgery should enable clinicians to develop strategies for effective risk reduction, such as minimizing the extent of surgery.

Based on the current literature data, the authors recommend pre-operative counseling as well as routine biochemical and clinical surveillance for hyperthyroidism during the post-operative period. Although diagnosis should be early and expectant, treatment should be symptomatic as allowed by spontaneous resolution of symptoms. Continued efforts to minimize the extent of PTH exploration surgery, if possible, may reduce the potential morbidity of thyrotoxicosis after surgery.

## Conflict of Interest Statement

The author declares that the research was conducted in the absence of any commercial or financial relationships that could be construed as a potential conflict of interest.
